# Supplementary figures and images for: Urinary Tract Infections Impair Adult Hippocampal Neurogenesis
Source: Biology (Basel). 2022 Jun 9;11(6):891. doi: 10.3390/biology11060891 (PMC9220213; doi:10.3390/biology11060891)

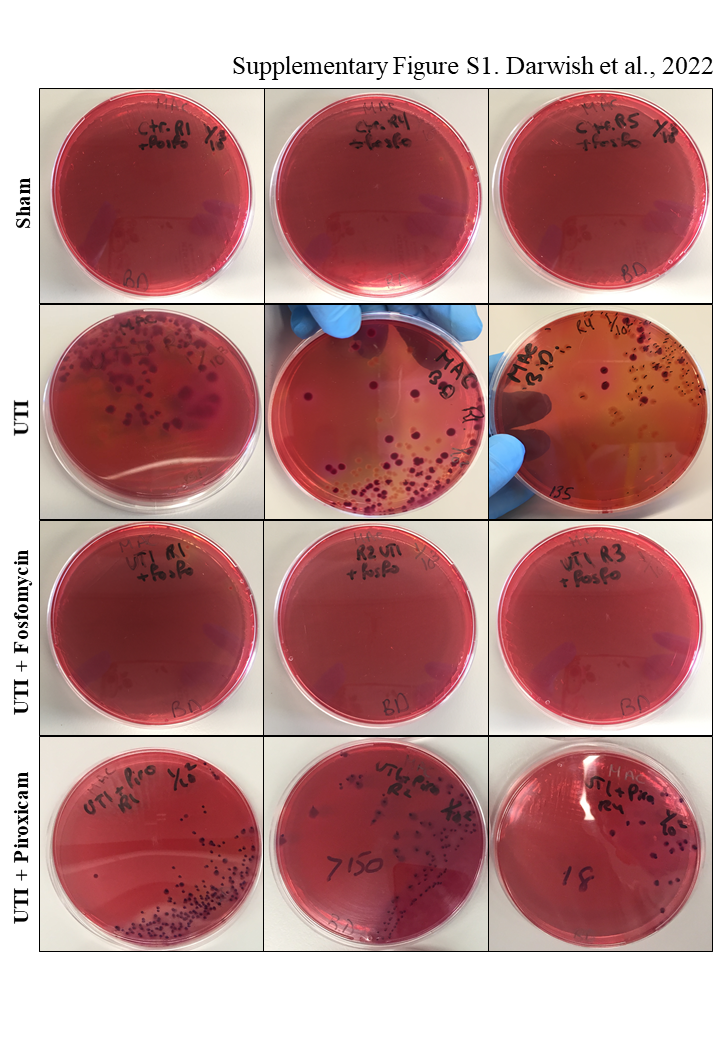

Supplement: Supplementary file 1 [file biology-11-00891-s001.zip › Supplementary Figure S1. Darwish et al. 2022.tif]

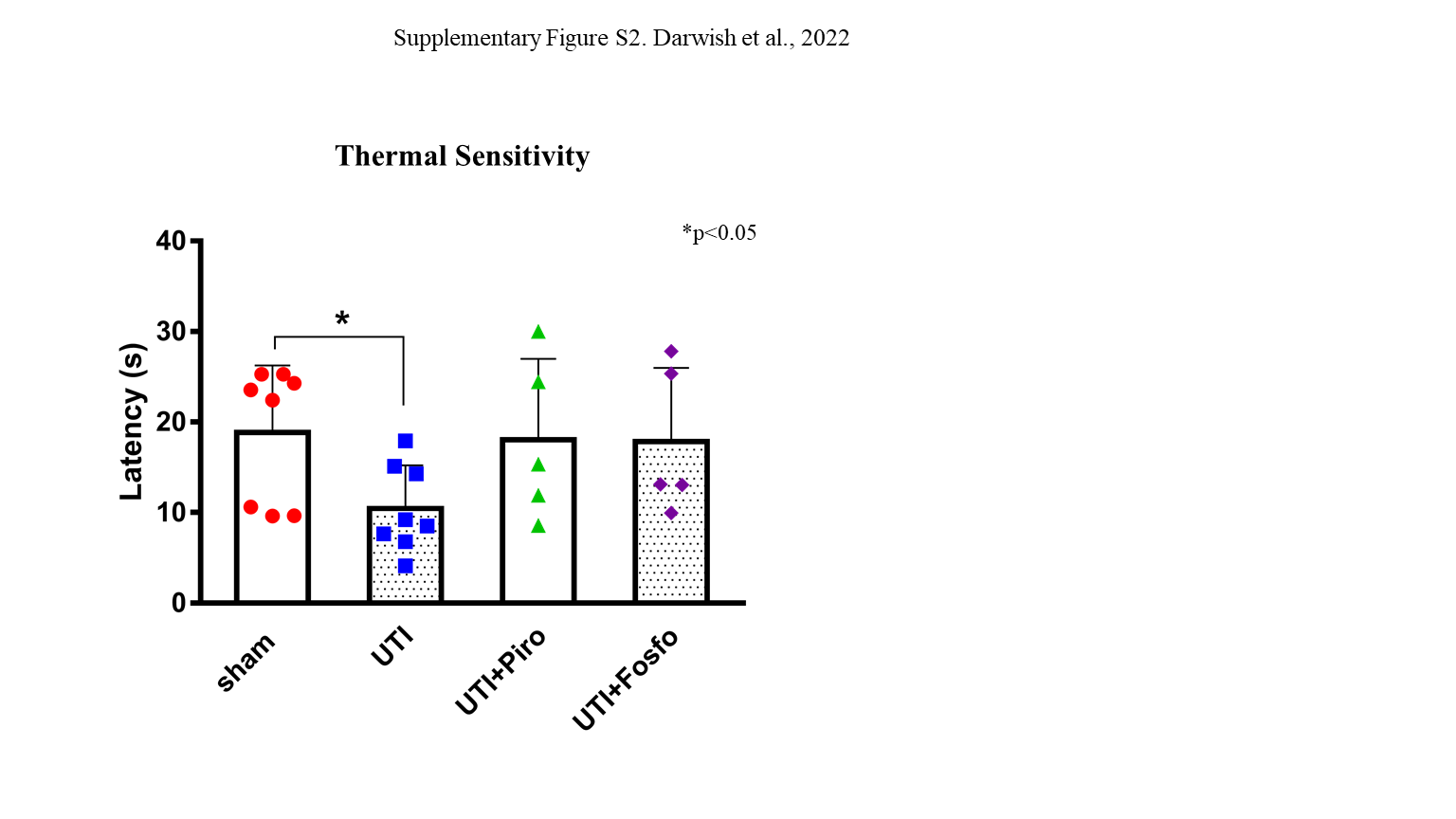

Supplement: Supplementary file 1 [file biology-11-00891-s001.zip › Supplementary Figure S2. Darwish et al. 2022.tif]

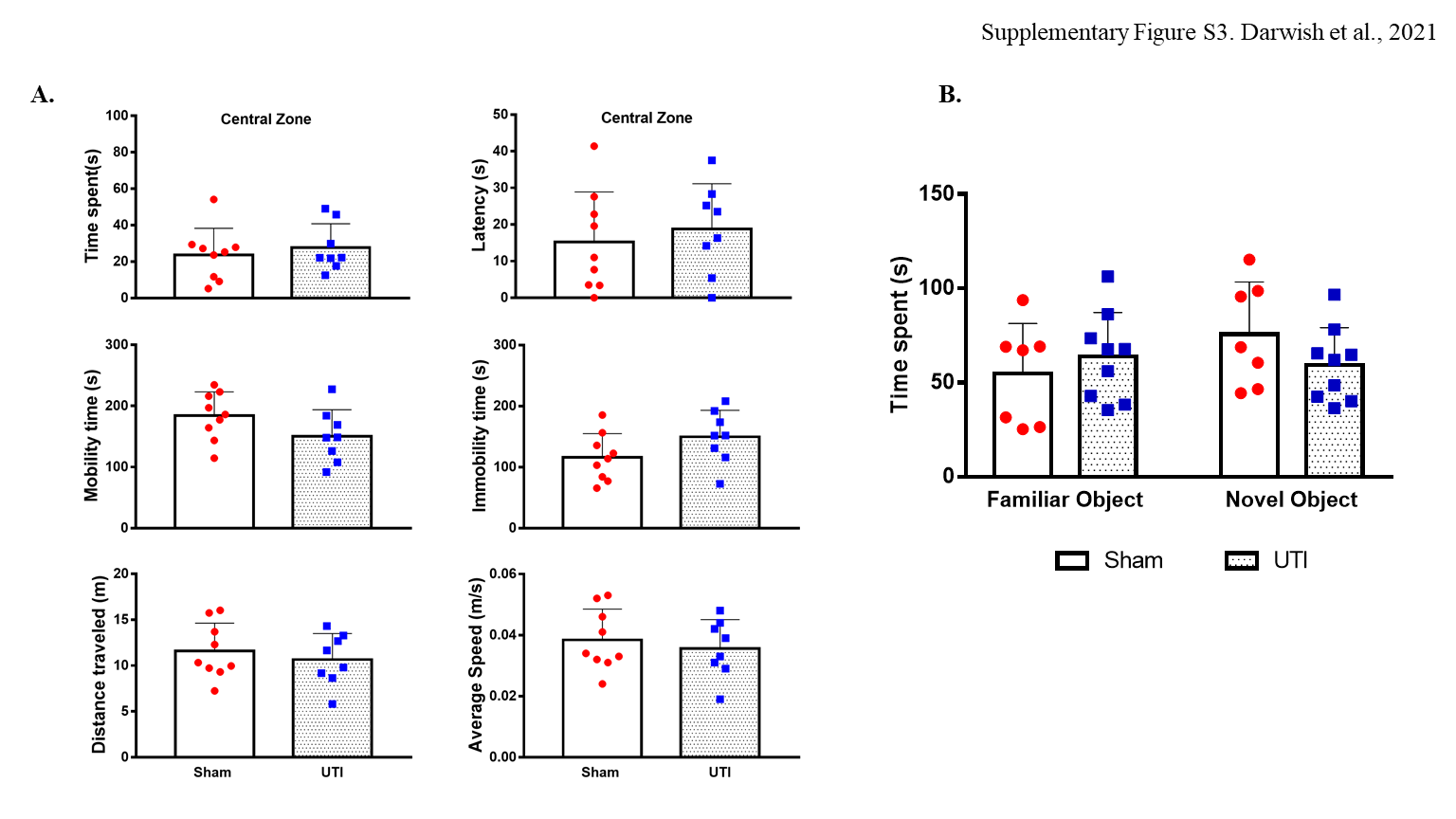

Supplement: Supplementary file 1 [file biology-11-00891-s001.zip › Supplementary Figure S3. Darwish et al. 2022.tif]
